# The small subunit of Rubisco and its potential as an engineering target

**DOI:** 10.1093/jxb/erac309

**Published:** 2022-07-18

**Authors:** Yuwei Mao, Ella Catherall, Aranzazú Díaz-Ramos, George R L Greiff, Stavros Azinas, Laura Gunn, Alistair J McCormick

**Affiliations:** SynthSys & Institute of Molecular Plant Sciences, School of Biological Sciences, King’s Buildings, University of Edinburgh, Edingburgh EH9 3BF, UK; SynthSys & Institute of Molecular Plant Sciences, School of Biological Sciences, King’s Buildings, University of Edinburgh, Edingburgh EH9 3BF, UK; SynthSys & Institute of Molecular Plant Sciences, School of Biological Sciences, King’s Buildings, University of Edinburgh, Edingburgh EH9 3BF, UK; School of Biological Sciences, University of Bristol, 24 Tyndall Avenue, Bristol BS8 1TQ, UK; Department of Cell and Molecular Biology, Uppsala University, S-751 24 Uppsala, Sweden; Department of Cell and Molecular Biology, Uppsala University, S-751 24 Uppsala, Sweden; Plant Biology Section, School of Integrative Plant Science, Cornell University, Ithaca, NY, USA; SynthSys & Institute of Molecular Plant Sciences, School of Biological Sciences, King’s Buildings, University of Edinburgh, Edingburgh EH9 3BF, UK; Western Sydney University, Australia

**Keywords:** Algae, carboxylation, oxygenation, photosynthesis, plants

## Abstract

Rubisco catalyses the first rate-limiting step in CO_2_ fixation and is responsible for the vast majority of organic carbon present in the biosphere. The function and regulation of Rubisco remain an important research topic and a longstanding engineering target to enhance the efficiency of photosynthesis for agriculture and green biotechnology. The most abundant form of Rubisco (Form I) consists of eight large and eight small subunits, and is found in all plants, algae, cyanobacteria, and most phototrophic and chemolithoautotrophic proteobacteria. Although the active sites of Rubisco are located on the large subunits, expression of the small subunit regulates the size of the Rubisco pool in plants and can influence the overall catalytic efficiency of the Rubisco complex. The small subunit is now receiving increasing attention as a potential engineering target to improve the performance of Rubisco. Here we review our current understanding of the role of the small subunit and our growing capacity to explore its potential to modulate Rubisco catalysis using engineering biology approaches.

## Teaser

A review of the role of the small subunit of Rubisco in the evolution, assembly, activity, and regulation of Rubisco, and engineering opportunities to enhance crop performance.

## Introduction

Carboxylation by Rubisco has been the dominant biological driving force for inorganic carbon sequestration since the early beginnings of life on our planet. In almost all prokaryotic and eukaryotic autotrophs, Rubisco catalyses the addition of CO_2_ to ribulose 1,5-bisphosphate (RuBP) to form two molecules of 3-phosphoglyceric acid (3PGA) as the initial step of inorganic carbon fixation through the Calvin–Benson–Basham (CBB) cycle (Raven, 2009). However, Rubisco can also catalyse the oxygenation of RuBP to generate 2-phosphoglycolate (2PG), a toxic metabolite that inhibits two enzymes in the CBB cycle (i.e. triose phosphate isomerase and sedoheptulose 1,7-bisphosphate phosphatase) and requires recycling back to 3PGA through the photorespiratory salvage pathway (Fernie and Bauwe, 2020). While photorespiration may play a regulatory role in carbon and nitrogen metabolism (Flügel *et al.*, 2017; Busch *et al.*, 2018; Busch, 2020; Timm, 2020; Shi and Bloom, 2021), it is generally considered an energetically wasteful process that results in the loss of previously fixed CO_2_, and reduces the overall efficiency of photosynthesis and crop yield potential (Zhu *et al.*, 2010). As such, Rubisco and the proteins associated with its assembly and regulation (the so-called ‘Rubiscosome’) have been key targets for crop improvement for several decades (Parry *et al.*, 2013; Erb and Zarzycki, 2018; Orr and Parry, 2020; von Caemmerer, 2020; Taylor *et al.*, 2022).

**Fig. 1.**
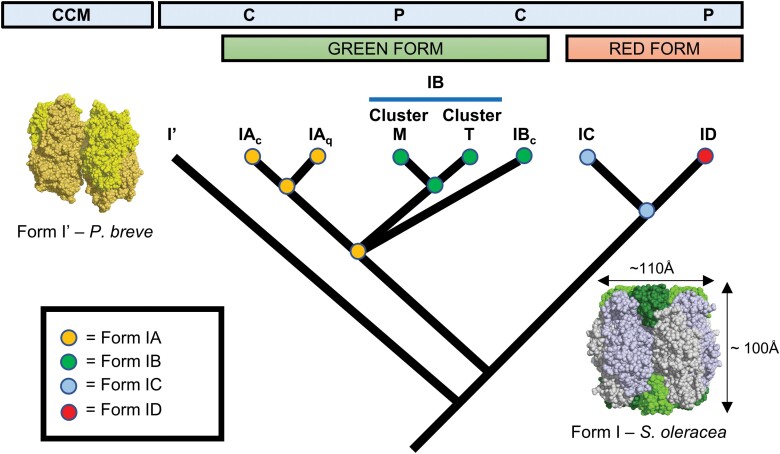
Evolutionary origins and diversification of Form I Rubisco. Differently coloured nodes represent the four broad types of Form I Rubisco, and their positions on the phylogram reflect currently hypothesized evolutionary trajectories. The biophysical carbon-concentrating mechanisms (CCMs) in which the various Form I Rubisco lineages take part are shown, with ‘C’ and ‘P’ signifying carboxysomal and pyrenoid-based mechanisms, respectively. Note, pyrenoid formation has been gained and lost several times in different organisms, so not all extant Form IB (M) and ID Rubisco molecules form pyrenoids. The representative crystal structures of Form Iʹ and I Rubisco are from *Promineofilum breve* [Protein Data Bank (PDB) code 6URA] and *Spinacia oleracea* (PDB: 1RCO), respectively.

Form I Rubisco is the dominant form found today and is considered the most abundant enzyme in the living world (Ellis, 1979; Tabita *et al.*, 2007; Bar-On and Milo, 2019; Hayer-Hartl and Hartl, 2020). It is characterized by the presence of eight small subunit (RbcS; ~12–18 kDa) protomers that hold together four large subunit (RbcL; ~50–55 kDa) dimers to form a hexadecameric L_8_S_8_ complex (~530–550 kDa) in the shape of a cylinder with a diameter and height of ~110 Å and 100 Å, respectively (Fig. 1) (Bracher *et al.*, 2017). As each RbcL dimer forms two active sites, RbcL has long been the primary focus of attempts to engineer improvements in Rubisco performance (for reviews, see Carmo-Silva *et al.*, 2015; Sharwood, 2017). In comparison, no RbcS residues interact directly with the active sites, and as such the RbcS has received relatively less attention. Although the RbcS is considered to play a structural role in stabilizing the L_8_ complex, experimental evidence has shown that the presence of the RbcS is also important for efficient assembly and maximal catalytic activity in all Form I Rubiscos (Morell *et al.*, 1997), expression of nuclear-encoded *rbcS* gene(s) (i.e. in most eukaryotes) plays a key role in regulating overall Rubisco levels (Rodermel, 1999; Wostrikoff and Stern, 2007; Wietrzynski *et al.*, 2021), and the composition of the RbcS peptide can have a significant impact on the catalytic parameters of the Rubisco complex (Box 1). Nevertheless, a comprehensive understanding of how the RbcS influences Rubisco activity remains elusive, and the structural role and functional importance of the RbcS in the evolution of Form I Rubisco are still far from fully understood (Banda *et al.*, 2020).

**Box 1. Common catalytic parameters of Rubiscos**

Net CO_2_ fixation by all Rubisco forms is determined by the difference between the rates of carboxylation and oxygenation, which are ultimately determined by the maximum rates of carboxylation (*V*_c_) and oxygenation (*V*_o_), the carboxylation turnover rate per active site (*k*_cat_^c^), the Michaelis–Menten constants for CO_2_ (*K*_c_ and *K*_c_^air^ in the absence and presence of O_2_, respectively), and O_2_ (*K*_o_), and the concentrations of CO_2_ and O_2_ at the Rubisco active site (Genkov and Spreitzer, 2009; Flamholz *et al.*, 2019). Further parameters include the specificity of Rubisco for CO_2_ versus O_2_, often called the specificity factor (*S*_c/o_ or Ω), which can be derived from the catalytic efficiency of carboxylation relative to the catalytic efficiency of oxygenation (i.e. *V*_c_*K*_o_/*V*_o_*K*_c_) (Parry *et al.*, 1989), Rubisco carboxylation efficiency (*k*_cat_^c^/*K*_c_^air^) (Orr *et al.*, 2016), and the initial response of the rate of carboxylation to the concentration of CO_2_ (*V*_c_/*K*_c_^air^) (Shih *et al.*, 2016).

Although Rubisco has been the subject of a considerable number of reviews over the past years, the last dedicated review for the RbcS was produced almost 20 years ago (Spreitzer, 2003), before techniques to modify RbcS genes in land plants were well established. Several excellent reviews have since discussed newer aspects of RbcS-related research (e.g. Bracher *et al.*, 2017; Sharwood, 2017), while advances in engineering biology [e.g. clustered regularly interspaced short palindromic repeats (CRISPR)/CRISPR-associated peptide (Cas)] have now made it possible to edit partial or entire RbcS gene families (Donovan *et al.*, 2020; Martin-Avila *et al.*, 2020; Matsumura *et al.*, 2020). Furthermore, the capacity to express plant Rubiscos in *Escherichia coli* has opened up new opportunities to screen RbcS families, ancestral forms of RbcS, and synthetic RbcS variants (Aigner *et al.*, 2017; Lin *et al.*, 2020, 2022). Although progress is being made, chloroplast engineering is still only well established in a small number of plant species (Ruf *et al.*, 2019; Yu *et al.*, 2020). Thus, strategies to engineer RbcS are arguably more advanced and may be easier to implement broadly in crop plants than those for RbcL. Developing our understanding of the extent to which the RbcS could enhance Rubisco performance should be a critical goal going forward in efforts to engineer improvements in photosynthetic capacity (Long *et al.*, 2019). This review is aimed as an update to Spreitzer (2003), and will include a discussion of our current understanding of the evolutionary significance of the RbcS, the role of RbcS in assembly, catalytic properties, and biophysical CO_2_-concentrating mechanisms, RbcS families in different plant species, and RbcS as an engineering target to enhance Rubisco performance (Long *et al.*, 2018; Atkinson *et al.*, 2020; Oltrogge *et al.*, 2020).

## The origins of Form I Rubisco and the small subunit

There are three known groups of Rubisco found in nature, Form I, II, and III, which differ in terms of structure and sequence (Tabita *et al.*, 2008), and a fourth group of Rubisco-like proteins (RLPs, or Form IV) that cannot catalyse carboxylation but instead function in various bacterial pathways, including sulfur metabolism and sugar degradation (Hanson and Tabita, 2001; Ashida *et al.*, 2003; Erb *et al.*, 2012; Zhang *et al.*, 2016). The most ancient of the three functional forms of Rubisco (Form III) could have emerged up to 3.5 billion years ago (bya) when the atmosphere was anoxygenic (Andersson and Backlund, 2008; Iñiguez *et al.*, 2020), and may have evolved from the enolase enzyme family in a non-CO_2_-fixing archaeal ancestor (Erb and Zarzycki, 2018). Form III Rubiscos are found mainly in anaerobic archaea and are typically associated with nucleotide and nucleoside metabolism rather than CO_2_ fixation through the CBB cycle, with a few exceptions (Frolov *et al.*, 2019). The discovery of Form II/III intermediates in the archaeal order Methanosarcinales has suggested that the original functional role of Rubisco may not have been to capture CO_2_, and that Rubisco-based autotrophy via the CBB evolved either in an ancient archaeon or later, possibly during the transfer of Form III-type Rubisco from archaea to eubacteria and the subsequent evolution of Form II and Form I Rubiscos (Tabita *et al.*, 2007; Wrighton *et al.*, 2016). The CBB cycle is speculated to have arisen subsequently from a primitive carbon metabolic pathway utilizing Rubisco, such as the archaeal reductive hexulose-phosphate pathway (Kono *et al.*, 2017; Erb and Zarzycki, 2018).

All functional Rubiscos share a common core structural component of two RbcL peptides that assemble head to tail into an L_2_ dimer to form two surface-exposed active sites, with each active site located at the interface of the C-terminal domain of one subunit and the N-terminal domain of the other. Form II Rubiscos can consist of one or more L_2_ dimers, while Form III Rubiscos are assembled in oligomeric arrays of 3–5 dimers (Kitano *et al.*, 2001). As both Form II and III Rubiscos lack RbcS, oligomerization must be facilitated by dimer–dimer interactions. For example, a 29 residue C-terminal structural domain in the Form III-type RbcL of the archaeon *Methanococcoides burtonii* acts as a small subunit ‘mimic’ that assists in the transition between dimeric and decameric states when substrate (i.e. RuBP) is present (Gunn *et al.*, 2017). Oligomerization probably acts to concentrate the Rubisco active sites and increases carboxylation efficiency, which is also considered one of the key contributions of the RbcS in Form I Rubisco. Based on the available catalytic data, extant Form II and Form III Rubiscos can be fast (i.e. have high *k*_cat_^c^ values), but both are highly sensitive to oxygen (O_2_), with *S*_c/o_ values ranging from 1 to 15 (Badger and Bek, 2008; Liu *et al.*, 2017).

Form I Rubiscos bearing RbcS most probably originated after the transfer of Form III Rubisco to eubacteria but prior to the divergence of proteobacteria and cyanobacteria following the evolution of oxygenic photosynthesis ~2.9 bya in the late Archaean (Nisbet *et al.*, 2007; Tabita *et al.*, 2007). Thus, the atmosphere would still have been rich in CO_2_ (Zang *et al.*, 2021). Phylogenetic analyses have indicated distinct periods of RbcL amino acid substitutions associated with adaptation to rising O_2_ stress during the transition from anaerobic Form III Rubisco to aerobic Form I Rubisco, which probably pre-date the acquisition of Form I Rubisco by fully derived cyanobacterial clades (Kacar *et al.*, 2017). Nevertheless, the subsequent global transition from a reducing to an oxidizing atmosphere during the so-called ‘Great Oxygenic Event’ (2.3–2.5 bya) was due to the success of early cyanobacteria that had acquired Form I Rubisco (Flamholz and Shih, 2020).

### The discovery of Form Iʹ Rubisco

The emergence of the RbcS is still an area of ongoing research and debate (Shih *et al.*, 2016). Recent work has uncovered a new clade of Form I-like, or Form Iʹ, Rubiscos in the non-phototrophic Anaerolineales order of the diverse Chloroflexi phylum that oligomerize into an octameric L_8_ complex, akin to the hexadecameric (L_8_S_8_) complex, but lack RbcS peptides (Fig. 1) (Banda *et al.*, 2020). This intriguing study highlights that RbcL can form octamers in the absence of RbcS via a fortified network of additional hydrogen bonds and salt bridges at the interface between L_2_ dimers. Although Form Iʹ may have evolved from a Form I Rubisco that subsequently lost its RbcS, the most parsimonious inference is that both Form I and Form Iʹ diverged from a common ancestor pre-dating cyanobacteria that lacked RbcS. Extant Form Iʹ Rubisco from the mesophilic Chloroflexi species ‘*Candidatus Promineofilum breve*’ has a low *S*_c/o_ value (36) compared with its close cyanobacterial relative *Synechococcus* sp. PCC 6301 (56), which has a typical RbcS-bearing Form I Rubisco. This suggests that the L_8_ Form Iʹ, like Form II and III, may have a limited capacity to evolve a higher specificity for CO_2_ to adapt to environments with elevated O_2_. However, the existence of a stable L_8_ core could have presented an opportunistic platform for the docking of ancestral RbcS-type peptides. Overall, the RbcS seems to have appeared as an evolutionary response to increasing O_2_ that afforded Form I Rubiscos a greater capacity to diversify their catalytic range (e.g. in terms of *k*_cat_^c^ and *S*_C/O_), while providing a scaffolding function to concentrate eight active sites (Spreitzer, 2003; Erb and Zarzycki, 2018). Modelling studies have also indicated that residues on the RbcS may bind CO_2_ and potentially act as a ‘reservoir’ to increase local CO_2_ availability, but this has yet to be experimentally verified (Van Lun *et al.*, 2014).

It remains unclear what kind of ancestral protein RbcS might have evolved from. The apparent sequence similarity between RbcS and CcmM35, a linker protein involved in Rubisco compartmentalization in cyanobacterial β-carboxysomes, has previously led to speculation that RbcS emerged from an early carboxysome-like bacterial microcompartment (Spreitzer, 2003). However, carboxysomes emerged a considerable time after Form I Rubisco evolved (Iñiguez *et al.*, 2020), and probably after the primary endosymbiotic event, as eukaryotic autotrophs do not appear to possess genes that code for carboxysome-like proteins (Shih *et al.*, 2016; Price *et al.*, 2019). More recent sequence analyses have indicated that the resemblance of CcmM35 to RbcS reflects small, coincidental local similarities rather than a close evolutionary relationship (Ryan *et al.*, 2019). CcmM35 also lacks key motifs used by RbcS to bind with RbcL. Cryo-EM analysis has confirmed that the RbcS-like domains of CcmM35 bind to Rubisco in a groove between two RbcL subunits and the adjacent RbcS, and do not use the RbcS-binding region or displace RbcS (Wang *et al.*, 2019; Zang *et al.*, 2021). Thus, it is likely that the RbcS has a different and possibly more ancient origin.

## RbcS diversity, structure, and interactions

**Fig. 2.**
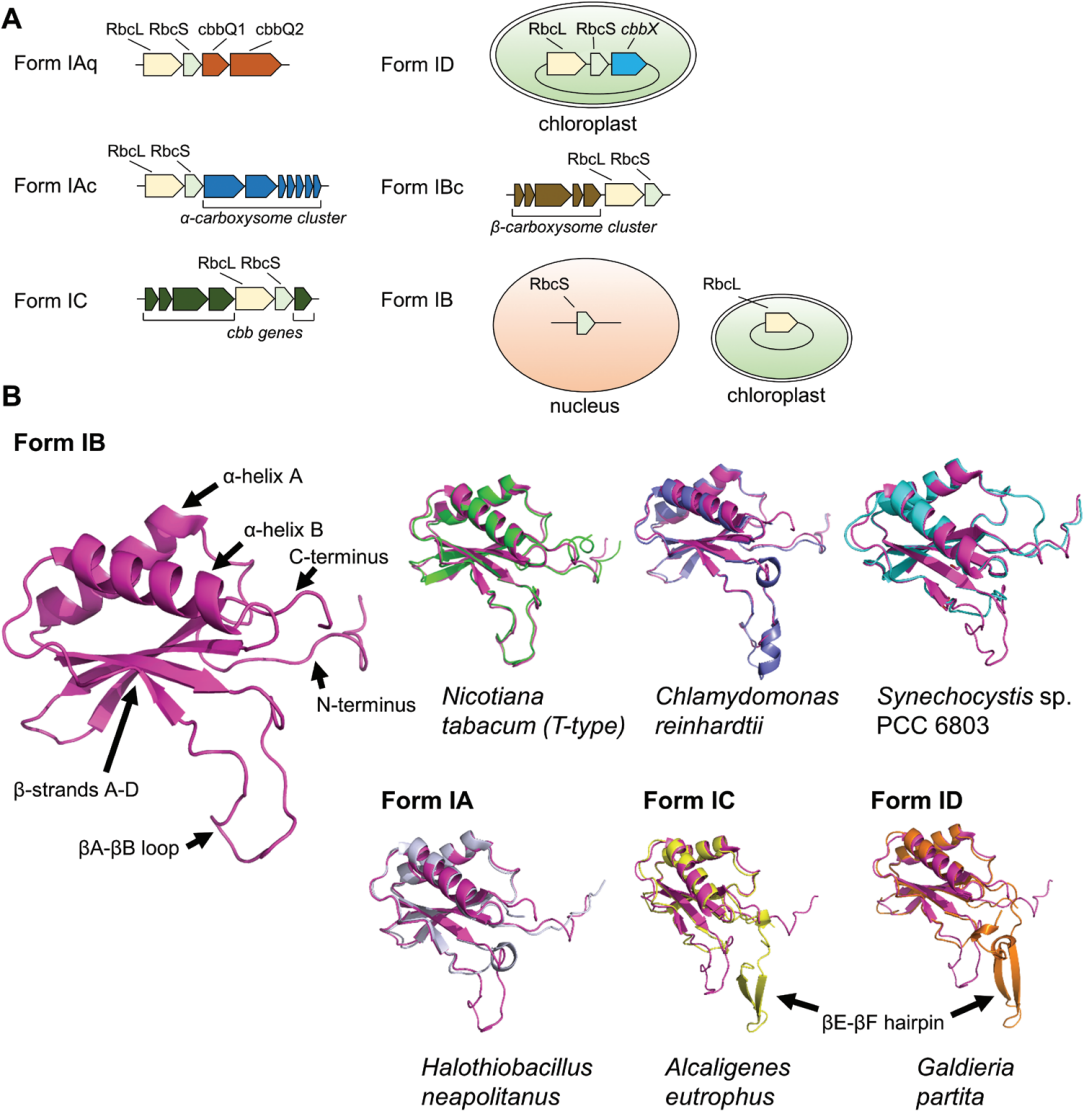
Gene maps and structural alignments of Form I Rubisco small subunits. (A) Gene maps of Form I Rubisco isoforms. Forms IAq, IAc, Ibc, and IC are encoded in bacterial genomic DNA on a single operon [adapted from Badger and Bek (2008); Multiple Rubisco forms in proteobacteria: their functional significance in relation to CO_2_ acquisition by the CBB cycle. Journal of Experimental Botany 59, 1525–154, by permission of the Society for Experimental Biology]. The Form ID Rubisco operon is located in the chloroplast genome. The Form IB Rubisco *RbcS* is nuclear encoded, while the *RbcL* is chloroplast encoded. (B) Superpositions of RbcS from *Nicotiana tabacum* (T-type NtRbcS-T) in green (predicted using AlphaFold) (Laterre *et al.*, 2017), *Chlamydomonas reinhardtii* (CrRbcS1) in purple (PDB: 1GK8), *Synechocystis* sp. PCC 6803 in cyan (AlphaFold code AF-P54206-F1), *Halothiobacillus neapolitanus* in grey (PDB: 1SVD), *Alcaligenes eutrophus* in yellow (PDB: 1BXN), and *Galdieria partita* in orange (1BWV) onto AtRbcS3B from *Arabidopsis thaliana* (*At*) in magenta (PDB: 5IU0). Arrows indicate secondary structural features.

Form I Rubiscos show considerable diversification and are divided into four subgroups consisting of the ‘green-types’ Forms IA and IB and ‘red-types’ Forms IC and ID (Fig. 2A) (Tabita *et al.*, 2008). Form IA is found in proteobacteria and cyanobacteria, and is further subdivided into Forms IAc and IAq based on differences in RbcS sequence and gene arrangements (Badger and Bek, 2008). The *RbcL* and *RbcS* genes for Form IAc Rubisco are found near an α-carboxysome operon, while Form IAq Rubisco genes are associated with the presence of the putative Rubisco chaperonin gene *cbbQ*, additional genes for Form II Rubisco, and the lack of an α-carboxysome operon. Form IB is the largest group and includes proteobacteria, cyanobacteria, green algae, and higher plants. It is subdivided into IB and IBc to indicate the Form IBc in cyanobacteria associated with β-carboxysomes (Badger *et al.*, 2002). Forms IC occurs in proteobacteria and the Chloroflexi, while Form ID can be found in proteobacteria and non-green algae, such as the Rhodophyta and Haptophyta. In prokaryotes, Form I Rubisco is found as an *RbcL–RbcS* operon, while in most eukaryotes *RbcL* is found on the chloroplast genome and *RbcS* has been transferred to the nuclear genome [with some exceptions, including the Rhodophyta and some Chromophyta (Valentin and Zetsche, 1989; Kostrzewa *et al.*, 1990)], and proliferated into an *RbcS* gene family.

The amino acid sequences of RbcL in Form I Rubiscos are relatively well conserved. For example, the RbcL sequences of Form IB Rubiscos in plants are generally 90% identical (Liu *et al.*, 2017). In contrast, RbcS are characterized by much greater sequence diversity, with only ≥30% similarity observed across different species (Bracher *et al.*, 2017). The core structure of each RbcL subunit is characterized by a short N-terminal domain consisting of a four-stranded β-sheet and two α-helices, and a longer C-terminal domain that forms an eight-stranded β/α-barrel (Whitney *et al.*, 2011; Valegård *et al.*, 2018). The conserved catalytic residues reside within the β/α-barrel, which form an active site together with residues from the N-terminal domain of the adjacent RbcL in each L_2_ dimer.

All RbcS also fold into a common core structure of a four-stranded antiparallel β-sheet, consisting of β-strands A–D, which are covered on one side by two α-helices (i.e. α-helices A and B) (Fig. 2B) (Knight *et al.*, 1990). The differences between RbcS isoforms in Forms IA–D Rubiscos include the length of the loop sequence between β-strand A and B (i.e. the βA–βB loop) and the length of the C-terminus, which in red-type Rubiscos contain an additional β hairpin consisting of β-strands E and F (i.e. the βE–βF hairpin) (Spreitzer, 2003; Andersson and Backlund, 2008). Furthermore, red-type Rubiscos can have a slightly longer loop between β-strands C and D (i.e. the βC–βD loop).

### The variability of the βA–βB loop

The βA–βB loop of each RbcS faces inwards towards the central solvent channel, or pore, formed by the (L_2_)_4_ assembly, while the two α-helices are solvent exposed. The length of the βA–βB loop is the most variable structural feature of the RbcS and is thought to regulate the width of the aperture of the solvent channel (Spreitzer, 2003). Prokaryotes and non-green algae are characterized by a short loop of only ~10 residues, plants have an average of 22 residues, while green algae tend to have longer βA–βB loops ranging from 20 to 31 residues. The length of the βA–βB loop has been used as a phylogenetic marker, for example to distinguish between Chlorophyte and Streptophyte algae (Goudet *et al.*, 2020). In red-type Rubiscos, which have RbcS with shorter βA–βB loops, the C-terminal βE–βF hairpins of the four RbcS on each end of the Rubisco complex come together to form a central β-barrel around the entrance of the solvent channel (Andersson and Backlund, 2008). This fills the space typically occupied by the βA–βB loop from green-type Rubiscos, although the contacts between the βA–βB loops of each RbcS in green-type Rubiscos are less extensive.

### Interactions between RbcS and RbcL

**Fig. 3.**
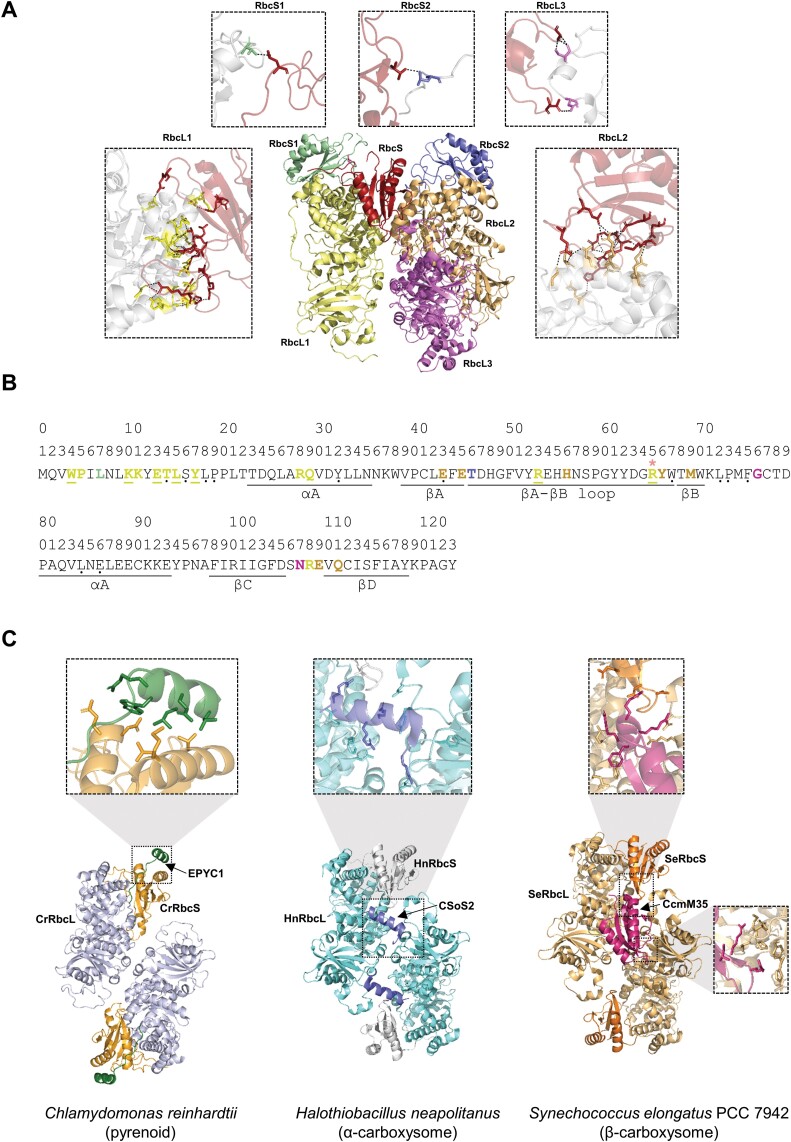
Interactions of the small subunit of Rubisco within the Rubisco complex and with Rubisco linker proteins. (A) Polar interactions of a given RbcS (shown in red) with adjacent subunits, including three RbcL (RbcL1, RbcL2, and RbcL3 shown in yellow, orange, and magenta, respectively) and two RbcS (RbcS1 and RbcS2 shown in green and blue, respectively) subunits are shown in an L_3_S_3_ representation of *Spinacia oleracea* Rubisco (PDB: 1RCO). Details of interactions with each subunit are shown in insets. (B) Sequence of SoRbcS1 from *Spinacia oleracea* with interacting residues highlighted [colours match subunits in (A)]. Highly and strictly conserved residues in higher plants are marked with a black dot or underlined, respectively. R65 interacts with both RbcL1 and RbcL3 (indicated with an asterisk, *). (C) Interactions between RbcS and RbcL with Rubisco linker proteins from *Chlamydomonas reinhardtii* (EPYC1, PDB: 7JFO), *Halothiobacillus neapolitanus* (CSoS2, PDB: 6UEW), and *Synechococcus elongatus* PCC 7942 (CcmM35, PDB: 6HBC), respectively, are shown using L_2_S_2_ representations (Wang *et al.*, 2019; He *et al.*, 2020; Oltrogge *et al.*, 2020). Inset views highlight the interfaces between EPYC1, CSoS2, and CcmM35 with Rubisco subunits. Interacting residues are listed in Table 1.

Each RbcS sits in the groove between two L_2_ dimers at the top and the bottom of the L_8_S_8_ complex and makes polar contacts with three RbcL and two neighbouring RbcS subunits (Fig. 3A, B; Table 1). However, for each RbcS, the majority of polar interactions occur with the β/α-barrel domains of two RbcL subunits, with the RbcS N-terminal region forming extensive interactions with one RbcL subunit that accounts for at least two-thirds of the overall interaction energy (Knight *et al.*, 1990; Ryan *et al.*, 2019). The second RbcL subunit interacts primarily with the βA–βB loop region of the RbcS. There are relatively fewer contacts with other subunits. For example, in spinach (*Spinacia oleracea*) Rubisco, two residues in the RbcS βC–βD loop interact with one of the RbcL N-terminal domains in a neighbouring L_2_ dimer, and L7 in the N-terminal domain and T46 in the βA–βB loop interact with an adjacent RbcS.

**Table 1.** Amino acid residue interactions with the small subunit of Rubisco

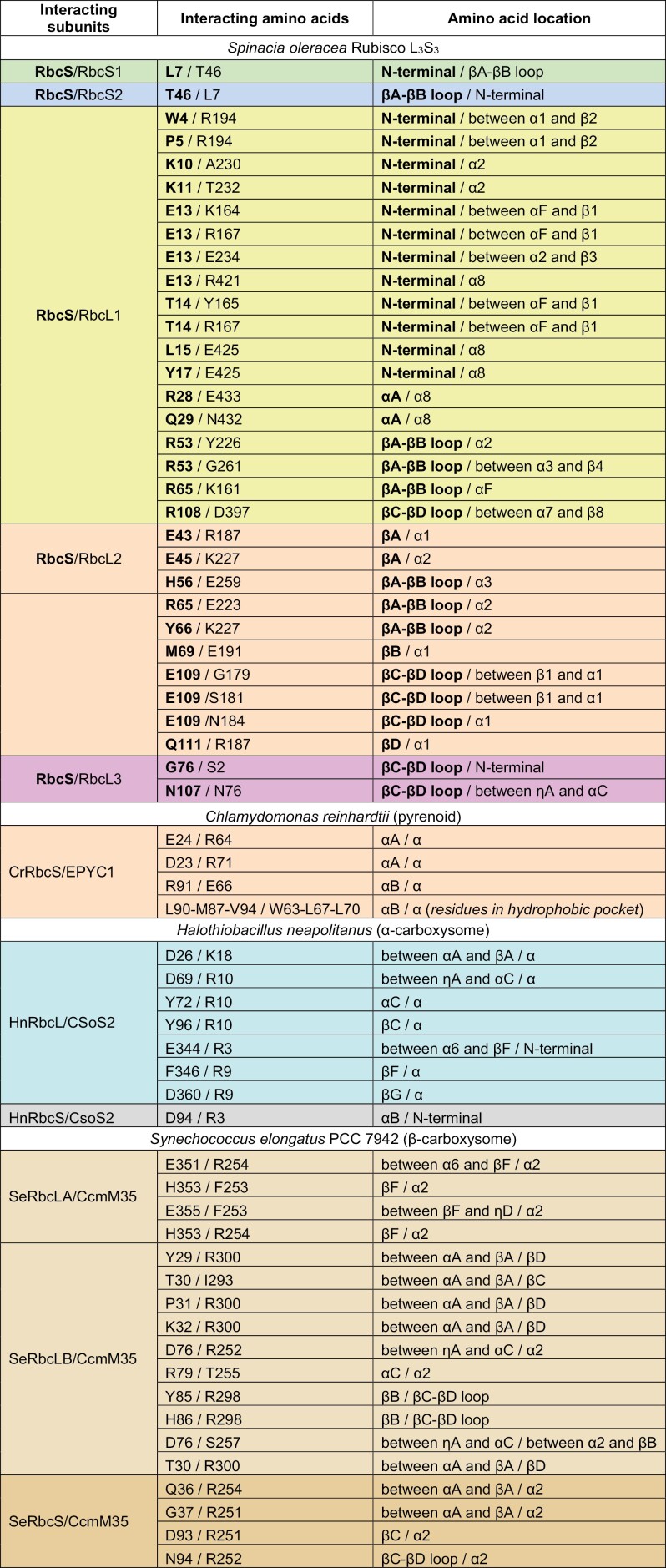

Interactions are shown for a given RbcS with adjacent subunits in the surrounding Rubisco complex from spinach (*Spinacia oleracea*, PDB: 1RCO) (Fig. 3A) and between RbcS or RbcL and the Rubisco linker EPYC1 from *Chlamydomonas reinhardtii* (PDB: 7JFO), CsoS2 from *Halothiobacillus neapolitanus* (PDB: 6UEW), and CcmM35 from *Synechococcus elongatus* PCC 7942 (PDB: 6HBC) (from Fig. 3C). Only polar interactions are shown between the RbcS and adjacent Rubisco subunits as determined using the InterfaceResidues.py script (https://pymolwiki.org/index.php/InterfaceResidues) in PyMOL (v.2.3.2). Interactions between the linker proteins and RbcS or RbcL include salt bridge, hydrogen bond, and cation-π interactions and, for EPYC1, residues involved in the formation of a hydrophic pocket. Amino acids and structural locations shown in bold represent the focal RbcS (in red in Fig. 3A); those not in bold represent the adjacent subunit. See Fig. 3 for matching illustrations and additional information.

**Table 2.** Recent engineering studies involving modifications to the small subunit of Rubisco

Background	RbcL	RbcS	Rubisco source and modification	Rubisco characteristics	Reference
*E. coli*	AtRbcL	AtRbcS3B	Arabidopsis	Similar catalytic properties of leaf Rubisco	Aigner *et al.* (2017)
	NtRbcL	NtRbcSA	Tobacco NtRbcS family	All M-type RbcS: similar to leaf RubiscoRbcS-T: *k*_cat_^c^↑28%; *K*_C_↑108%	Lin *et al.* (2020)
	TeRbcL	TeRbcS	*Thermosynechococcus elongatus* BP1 with PMs in RbcL and RbcS	TeRbcL (P415A)/TeRbcS (V98M):*k*_cat_^c^↑28%; *K*_c_↓13%; k_cat_^c^/K_c_^air^↑42%; S_C/O_↑6%	Wilson *et al.* (2018)
*Chlamydomonas reinhardtii *(RbcS KO mutant rbcSΔ-T60-3)	CrRbcL	CrRbcS1	Native RbcS with vascular plant or *Synechococcus elongatus PCC *7942 βA–βB loop	Spinach: *V*_c_↓51%; *K*_c_↓26%; *K*_o_↓14%; *S*_C/O_≈*Synechococcus*: *V*_c_↓46%; *K*_c_≈; *K*^o^↓30%; *S*_C/O_↓11%	Karkehabadi *et al.* (2005)
	CrRbcL	CrRbcS1	Native RbcS with plantβA–βB loop and five RbcL PMs	*k*_cat_^c^↓44%; *V*_c_↓45%; *K*_c_↑26%; *V*_o_↓42%; *K*_o_↓12%; *S*_c/o_↑12%	Spreitzer *et al.* (2005)
	CrRbcL	CrRbcS1	Native RbcS with various PMs	*V*_c_↓63-92%; *K*_c_↑0-207%; *K*_o_↑0-208%; *S*_C/O_↓0-9%	Genkov and Spreitzer (2009)
	CrRbcL	SoRbcSAtRbcS1BHaRbcS	Hybrid Rubiscos with plant RbcS	Spinach: *V*_c_↓13%; *K*_c_≈; *K*_o_↑8%; *S*_C/O_↑7%Arabidopsis: *V*_c_↓6%; *K*_c_≈; K_o_↑10%; *S*_C/O_↑11%Sunflower: *V*_c_≈; *K*_c_≈; *K*_o_↑12%; *S*_C/O_↑3%	Genkov *et al.* (2010)
	CrRbcL	SoRbcS	Spinach RbcS with Chlamydomonas α-helices	*V*_c_↓88%; *K*_c_↑17%; *K*_o_↓43%; *S*_c/o_↓11%	Meyer *et al.* (2012)
	CrRbcL	CrRbcS1	Chlamydomonas RbcS with PM: I58W3	*V*_c_↓73%; *K*_c_↑43%; *K*_o_↑133%; *S*_C/O_↓15%	Esquivel *et al.* (2013)
	CrRbcL	NtRbcS-S1	Hybrid Rubisco with tobacco RbcS	*V*_c_↓22%; *K*_c_↓21%; *K*_o_↓14%; *S*_C/O_↑8%	Wachter *et al.* (2013)
	CrRbcL	NtRbcS-S1 NtRbcS-Tc	Tobacco M-type or T-type RbcS	NtRbcS-S1 (M-Type): *V*_c_↓13%; *K*_c_↓24%NtRbcS-Tc (T-type): *V*_c_↑7%; *K*_c_↑9%	Laterre *et al.* (2017)
*Arabidopsis thaliana (Col-0)*	AtRbcL	PsRbcS3A	Wild type with pea RbcS	Pea RbcS=15–18% of total RbcS pool; carboxylase activity↓11%	Getzoff *et al.* (1998)
	AtRbcL	AtRbcS	*1a3b* mutant	RbcS transcript pool↓80%; Rubisco content↓63%; FW↓81%; A_amb_↓65%	Izumi *et al.* (2012)
	AtRbcL	CrRbcS2	*1a3b* mutant with Chlamydomonas RbcS	*k*_cat_^c^↓12%;*K*c≈; *K*_c_^air^≈; *k*_cat_^c^/*K*_c_^air^≈; *S*_c/o_↓5%; Rubisco content↑117%; FW↑463%; *V*_cmax_↑52%	Atkinson *et al.* (2017)
	AtRbcL	AtRbcS	*1a2b3b* mutant	Rubisco content↓97%; FW↓99%; *V*_cmax_↓90%	Khumsupan *et al.* (2020)
*Nicotiana tabacum*(cv. Samsung, NC89 or Petite Havana)	NtRbcL	NtRbcS	OE of native RbcS in plastid	Plastid RbcS assembled into Rubisco, but only represented ~1% of total RbcS pool	Whitney and Andrews (2001)
	NtRbcL	NtRbcS	OE of native RbcS in plastid. KD of nuclear RbcS	RbcS protein pool≈; *A*_amb_↓22%	Dhingra *et al.* (2004)
	NtRbcL	NtRbcS	Plastid-expressed linked RbcL–RbcS	*k*_cat_^c^≈%; *K*_c_↓15%; *K*_o_↓45; *S*_c/o_≈; Rubisco content↓20%	(Whitney *et al.*, 2009)
	LeRbcL	NtRbcS	Tomato RbcL andnative RbcS family	*k*_cat_^c^↑27; Rubisco content↓; DW↓23%; *V*_cmax_↓45%	Zhang *et al.* (2011)
	NtRbcL	NtRbcS	CRISPR repression of native RbcS	Rubisco content↓93%; FW↓92%; *V*_cmax_↓58%; *A*_amb_↓57%	Donovan *et al.* (2020)
	SeRbcL	NtRbcS	*S. elongatus* PCC 7942 RbcL, CcmM35 and native RbcS	*V*_c_↑154%; *K*_c_↑1444%; *V*_o_↓64%; *K*_o_↑47%; *S*_c/o_↓33%Rubisco content↓91%; Growth↓; *A*_amb_↓	Orr *et al.* (2020)
	StRbcL	NtRbcS1StRbcS1StRbcS2StRbcS3StRbcS-T	Potato RbcL with native RbcS (nuclear), or potato RbcS1, RbcS2, RbcS,3 or RbcS-T expressed in plastid	StRbcL/NtRbcS: *k*_cat_^c^≈;*K*_c_≈; *k*_cat_^c^/*K*_c_^air^≈; *S*_c/o_≈; Rubisco content≈StRbcL/StRbcS1: *k*_cat_^c^≈; *K*_c_≈; *k*_cat_^c^/*K*_c_^air^≈; *S*_c/o_↑5%; Rubisco content↓55%StRbcL/StRbcS2: *k*_cat_^c^≈; *K*_c_≈; *k*_cat_^c^/*K*_c_^air^≈; *S*_c/o_≈; Rubisco content↓65%StRbcL/StRbcS3: *k*_cat_^c^↑13%; *K*_c_≈; *k*_cat_^c^/*K*_c_^air^↑7%; *S*_c/o_↑8%; Rubisco content↓73%StRbcL/StRbcS-T: *k*_cat_^c^↑42%; *K*_c_↑174%; *k*_cat_^c^/*K*_c_^air^↓49%; *S*_c/o_↓12%; Rubisco content↓63%	Martin-Avila *et al.* (2020)
*Oryza sativa*(cv. Notohikari, cv. Nipponbare, or subsp. *indica* cv. IR64)	OsRbcL	OsRbcS	OE of native RbcS2	RbcS transcript pool↑166%; Rubisco content↑52%; Activity↑60%; *A*_amb_≈	Suzuki *et al.* (2007)
	OsRbcL	SbRbcS	Sorghum RbcS	*k*_cat_^c^↑48%; *K*_c_↑39%; *S*_c/o_↓12%; Rubisco content↑24%; Activation↓11%; Growth≈; *V*_cmax_↑31%	Ishikawa *et al.* (2011)
	OsRbcL	OsRbcS	OE of nativeT-type RbcS	*k*_cat_^c^↑46%; *K*_c_↑200%; *S*_c/o_↓9%; Rubisco content↑21%; Activation↓32%; DW↓23% *V*_cmax_↑117%	Morita *et al.* (2014)
	OsRbcL	SbRbcS	Sorghum RbcS andKO of native RbcS family	*k*_cat_^c^↑79%; *K*_c_↑91%; *K*_c_^air^↑107%; *k*_cat_^c^/*K*_c_^air^↑15%; *S*_c/o_↓14%; Rubisco content↓56%; Activation↑42%; DW↓57%; *V*_cmax_≈	Matsumura *et al.* (2020)
	OsRbcL	OsRbcS	KD of native RbcS2 and RbcS4	Rubisco content↓40%; DW↓28%	Maheshwari *et al.* (2021)
	OsRbcL	OsRbcS	OE of native RbcS2	RbcS transcript pool↑130%; Rubisco content↑28%; DW↑23%	Yoon *et al.* (2020)
	OsRbcL	OsRbcS2	OE of native RbcS2 and RCA	Rubisco content↑44%; Activation≈; *V*_cmax_≈; *A*_amb_↑21% at 36°C	Suganami *et al.* (2021)
*Zea mays* (cv. Hi-II)	ZmRbcL	ZmRbcS	OE of nativeRbcS, RbcL, and RAF1	RbcS-RAF1: Rubisco content↑40%; Activation↓25%; Activity↑37; FW↑30%; *V*_cmax_≈RbcSL-RAF1: Rubisco content↑35%; Activation↓16%; Activity↑47% FW↑27%; *V*_cmax_≈	Salesse-Smith *et al.* (2018)

Impacts on the catalytic parameters of Rubisco, Rubisco content, activity, and activation state, and subsequent changes in growth and physiological phenotype are shown where data were available. For plant studies, arrows indicate change relative to the host background. For *C. reinhardtii*, changes are shown in comparison with the wild-type strain. For *E. coli*, changes are shown in comparison with measurements of Rubisco from the native host. Rubisco catalytic parameters are defined in Box 1. See Spreitzer (2003) for earlier work. Abbreviations/symbols: ≈, no significant difference; *A*_amb_, CO_2_ assimilation rate at ambient CO_2_ concentrations; *V*_cmax_, maximum achievable rate of Rubisco carboxylation; PM, point mutation; OE, overexpression; KD, knockdown; KO, knockout; At, Arabidopsis; Nt, tobacco; Te, *Thermosynechococcus elongatus* BP1; Cr, Chlamydomonas; So, spinach; Ha, sunflower; Ps, pea; Le, tomato; Se, *Synechococus elongatus* PCC 7942; St, potato; Os, rice; Sb, *Sorghum bicolor*; Zm, maize

**Table 3.** Examples of Rubisco small subunit gene families and their relative transcript abundances

Species	RbcS isoforms	Gene name	Gene accession	Relative abundance	RbcS Type-T expression	References
*Chlamydomonas *	2	CrRbcS1	Cre02.g120100	44.7		Fang *et al.* (2012)
*reinhardtii*		CrRbcS2	Cre02.g120150	53.3		
*Arabidopsis*	4	AtRbcS1A	AT1G67090	78.7		Mergner *et al.* (2020)
*thaliana*		AtRbcS1B	AT5G38430	1.4		
		AtRbcS2B	AT5G38420	8.2		
		AtRbcS3B	AT5G38410	11.7		
*Glycine *	3	GmRbcS1	GLYMA_19G046600	42.5		PhytoMine dataset
*max*		GmRbcS2	GLYMA_19G046800	57.4		
		GmRbcS-T	*GLYMA_13G097100*	0.1	Nodules, root	
*Nicotiana *	13	NtrbcS-S1a	KM025316.1	22.9		Sierro *et al.* (2014)
*tabacum*		NtrbcS-S1b	KM025317.1	19.9		
		NtrbcS-S2	KM025319.1	10.6		
		NtrbcS-S3	KM025321.1	1.6		
		NtrbcS-S4	KM025323.1	0.1		
		NtrbcS-S5	KM025325.1	3.1		
		NtrbcS-T1	KM025327.1	40.5		
		NtrbcS-T2	KM025329.1	0.7		
		NtrbcS-T3a	KM025331.1	0.0		
		NtrbcS-T4a	KM025334.1	0.7		
		NtrbcS-T5	KM025337.1	0.7		
		NtrbcS-T	*DV157962.1*	0.0	Trichome	
*Oryza *	5	OsRbcS1	*Os02g0152400*	0.0	Embryo, seed, pistil	Davidson *et al.* (2012)
*sativa*		OsRbcS2	Os12g0274700	50.3		
		OsRbcS3	Os12g0291100	41.1		
		OsRbcS4	Os12g0292400	2.8		
		OsRbcS5	Os12g0291400	5.8		
*Solanum *	6	SlRbcS1	Solyc02g063150.3	19.7		Tomato Genome Consortium (2012)
*lycopersicum*		SlRbcS2	Solyc03g034220.3	55.5		
		SlRbcS3A-C	Solyc02g085950.3	24.8		
		Sly-t	*Solyc07g017950.3*	0.0	Epidermis, fruit, bud	
*Solanum *	5	StRbcS1/pS1	PGSC0003DMG400019584	56.4		Potato Genome Sequencing Consortium (2011)
*tuberosum*		StRbcS2B/pS2	PGSC0003DMG400026409	6.7		
		StRbcS2C	PGSC0003DMG400012666	5.8		
		StRbcSC/ps3	PGSC0003DMG400024182	31.1		
		Stu-t/pST	*PGSC0003DMG400004097*	0.0	Stamen, flower	
*Triticum *	25	TaRbcS1A	TraesCS2A02G066700	2.9		Wheat Expression Browser
*aestivum*		TaRbcS2A	TraesCS2A02G066800	5.0		
		TaRbcS3A	TraesCS2A02G066900	1.5		
		TaRbcS4A	TraesCS2A02G067000	0.4		
		TaRbcS5A	TraesCS2A02G067100	0.8		
		TaRbcS6A	TraesCS2A02G067200	1.3		
		TaRbcS7A	TraesCS2A02G067300	1.6		
		TaRbcS8A	TraesCS5A02G165400	10.3		
		TaRbcS9A	TraesCS5A02G165700	11.9		
		TaRbcS1B	TraesCS2B02G078900	0.9		
		TaRbcS2B	TraesCS2B02G079100	1.1		
		TaRbcS3B	TraesCS2B02G079200	2.5		
		TaRbcS4B	TraesCS2B02G079300	2.2		
		TaRbcS5B	TraesCS2B02G079400	1.1		
		TaRbcS6B	TraesCS2B02G079500	2.6		
		TaRbcS7B	TraesCS5B02G162600	6.4		
		TaRbcS8B	TraesCS5B02G162800	9.3		
		TaRbcS1D	TraesCS2D02G065100	3.2		
		TaRbcS2D	TraesCS2D02G065200	3.6		
		TaRbcS3D	TraesCS2D02G065300	2.2		
		TaRbcS4D	TraesCS2D02G065400	1.2		
		TaRbcS5D	TraesCS2D02G065500	0.0		
		TaRbcS6D	TraesCS2D02G065600	2.4		
		TaRbcS7D	TraesCS5D02G169600	10.7		
		TaRbcS8D	TraesCS5D02G169900	14.9		
*Sorghum bicolor*	1	SbRbcS1	SORBI_3005G042000	100		Davidson *et al.* (2012)
*Zea*	2	RbcS1	GRMZM2G098520	76.9		NAM Consortium
*mays*		RbcS2	GRMZM2G113033	23.1		

Only RbcS isoforms with verified gene expression are shown. Italicized gene accessions indicate cluster T-type RbcS isoforms (Laterre *et al.*, 2017). For *Solanum lycopersicum*, RbcS3A, RbcS3B, and RbcS3C are represented by the same accession number. Gene names for *Glycine max*, *Triticum aestivum*, and *Sorghum bicolor* have been assigned based on genomic location. RNA-seq data were obtained from Expression Atlas (https://www.ebi.ac.uk/gxa/home). For Chlamydomonas, *G. max*, *N. tabacum*, and *T. aestivum* RNA-seq data were obtained from Fang *et al.* (2012), PhytoMine (https://phytozome-next.jgi.doe.gov/phytomine/begin.do), Sierro *et al.* (2014), and Wheat Expression Browser (http://www.wheat-expression.com), respectively. Relative abundance refers to the percentage contribution of each RbcS gene to the total RbcS transcript pool for each species

### The role of RbcS in biophysical CO_2_-concentrating mechanisms

RbcS is also an important component in the assembly of Rubisco-containing micro-compartments associated with biophysical CO_2_-concentrating mechanisms (CCMs), namely the carboxysomes in cyanobacteria and pyrenoids in algae and hornworts (see recent reviews by Hennacy and Jonikas, 2020; Barrett *et al.*, 2021; Borden and Savage, 2021). In both α- and β-carboxysomes, condensation of Rubisco by liquid–liquid phase separation appears to play a key part in carboxysome biogenesis (Wang *et al.*, 2019; Oltrogge *et al.*, 2020; Zang *et al.*, 2021). Condensation is mediated by low affinity, multivalent interactions between Rubisco and the linker proteins CsoS2 and CcmM35 in α- and β-carboxysomes, respectively. Both proteins interact with residues on RbcL and RbcS, either by intrinsically disordered, linear motifs in CsoS2 or by folded domains in CcmM35 (Fig. 3C; Table 1). Similarly, condensation of the Rubisco matrix within the pyrenoid of the green alga *Chlamydomonas reinhardtii* (hereafter Chlamydomonas) is mediated by the linker protein EPYC1 via five conserved motifs that interact exclusively with the α-helices of the RbcS (Mackinder *et al.*, 2016; Atkinson *et al.*, 2019; He *et al.*, 2020). This motif is a common feature of many other proteins found within the Rubisco matrix, which indicates that the RbcS plays a key role in mediating pyrenoid assembly in Chlamydomonas (Meyer *et al.*, 2020). Nevertheless, sequences of the RbcS α-helices differ significantly between species with pyrenoids (Goudet *et al.*, 2020), and EPYC1 is not broadly conserved. This suggests that the peptide sequences required for interactions between Rubisco and linker proteins are highly variable, which may relate to the wide diversity of different pyrenoid architectures and that pyrenoids appear to have evolved several times (Villarreal and Renner, 2012; Barrett *et al.*, 2021).

### Putative RbcS chaperones and the phenomenon of RbcS homogeneity in Rubisco

Our present understanding of the ancillary chaperones required for RbcL folding and Form I Rubisco assembly in prokaryotes and eukaryotes has been reviewed in detail recently (see Bracher *et al.*, 2017; Hayer-Hartl and Hartl, 2020). In prokaryotes, current models show that the (L_2_)_4_ assembly is held in place by the chaperones RbcX and RAF1, which are then displaced by RbcS to form the L_8_S_8_ complex (Xia *et al.*, 2020). In eukaryotes, an additional step is involved, where the stromal protein BSD2 displaces RbcX and RAF1, and in turn is displaced by RbcS (Aigner *et al.*, 2017). The mechanisms regulating RbcS binding and chaperone displacement are not yet fully understood. Unlike RbcL, RbcS is able to fold spontaneously and remains monomeric when expressed heterologously in *E. coli* (Andrews and Ballment, 1983; Andrews, 1988), but *in vivo* RbcS folding might require chaperones. In eukaryotes, RbcS may require assistance for folding following import and maturation into the chloroplast, possibly by the stromal chaperone Hsp70 (Wilson *et al.*, 2019). In some plant species, the N-terminal methionine of the mature RbcS peptide is methylated, although the functional significance remains unclear (Ying *et al.*, 1999). Furthermore, evidence in maize (*Zea mays*) and *Arabidopsis thaliana* (hereafter Arabidopsis) suggests that RbcS might form transient complexes with RAF1, RAF2, and BSD2, which in turn facilitate docking of RbcS with the (L_2_)_4_ assembly (Feiz *et al.*, 2014; Fristedt *et al.*, 2018). Notably, the genomes of organisms with red-type Rubisco lack several assembly chaperones, such as RbcX and RAF1, and instead rely on the plastid-encoded RbcS for assembly, with the C-terminal β-hairpin extension of the red-type RbcS playing a critical role (Joshi *et al.*, 2015). It is possible that the red-type RbcS may more closely represent the ancestral form of RbcS prior to the evolution of additional assembly chaperones.

In eukaryotes with green-type Rubiscos, the composition of RbcS isoforms in each Rubisco complex *in vivo* is still unclear (Yamada *et al.*, 2019). One intriguing phenomenon, based on current empirical evidence, is that eukaryotic Rubiscos may assemble with only one RbcS isoform per L_8_S_8_ complex, despite the presence of a family of different nuclear-encoded RbcS isoforms in most species. Multiple examples of crystal structures exist for plant Rubiscos, with eight identical RbcS isoforms, albeit with a few potential exceptions (Shibata *et al.*, 1996; Loewen *et al.*, 2013). More recently, Valegård *et al.* (2018) described a crystal structure for Arabidopsis Rubisco that was homogenous for the low abundance isoform RbcS1B, which represents only 3–5% of the total RbcS pool (Khumsupan *et al.*, 2020). Structural characterization of Rubisco using alternative approaches (e.g. cryo-EM) should help to elucidate if such findings are an artefact of the crystallization process, or biologically relevant. If the Rubisco assembly process does favour one RbcS isoform per L_8_S_8_ complex, this raises interesting questions about the potential mechanisms involved. One possibility is that binding of the first monomeric RbcS protomer could modify the structural conformation of the (L_2_)_4_ assembly to strongly favour the subsequent addition of the same isoform type. Although hypothetical, such a scenario could offer an efficient means of regulating the catalytic properties of the Rubisco pool through the expression of different RbcS isoforms.

## Influence of the RbcS on the assembly and catalytic properties of Rubisco

The efficiency of CO_2_ assimilation by Rubisco appears to be constrained by several catalytic trade-offs. Mechanistic models have hypothesized an apparent inverse relationship between *k*_cat_^c^ and *S*_c/o_, or that any improvement in carboxylation efficiency may also improve oxygenation efficiency (for a more detailed, review see Flamholz *et al.*, 2019). Several authors have argued that Form I Rubiscos may now be optimized to their existing environments, having reached a ‘Pareto optimality’ of activity and specificity in which neither parameter can be further improved without negatively affecting overall fitness (Tcherkez *et al.*, 2006; Savir *et al.*, 2010; Erb and Zarzycki, 2018). Nevertheless, new lines of evidence now suggest that Rubisco may have more room to manoeuvre than earlier thought. Firstly, previous arguments have been based on data from a relatively small pool of organisms. More expanded analyses have indicated that Rubisco has a greater degree of catalytic variability than expected and that the apparent catalytic trade-offs observed for Form I Rubiscos are weaker when larger groups are considered (Young *et al.*, 2016; Flamholz *et al.*, 2019). Bouvier *et al.* (2021) have also argued that the evolution of Rubisco in plants has been limited more by phylogenetic constraints than potential catalytic trade-offs. Such constraints may include the necessity for Rubisco to exhibit high levels of expression and protein stability, and a requirement to maintain complementarity with chaperones involved in assembly and regulation (i.e. the native Rubiscosome). Secondly, engineering efforts have screened predicted ancestral Rubiscos and produced Rubisco variants that deviate from the ‘canonical’ catalytic trade-offs between *k*_cat_^c^, *K*_c_, and *S*_c/o_ (Wilson *et al.*, 2018; Zhou and Whitney, 2019; Martin-Avila *et al.*, 2020; Lin *et al.*, 2022). Together, these studies suggest a wider scope for engineering improvements in the catalytic parameters of Rubisco, and that furthering our understanding of the underlying structural basis that confers the catalytic properties of Rubisco is still critically important (Valegård *et al.*, 2018).

In the context of the RbcS, it has been clear for decades that it influences assembly and the catalytic performance of the Form I Rubisco complex, and earlier work has been well reviewed (e.g. Spreitzer, 2003). For example, even in rare cases in cyanobacteria where RbcL can assemble in the absence of RbcS, the L_8_ complex exhibited only 0.15% of the carboxylase activity of the L_8_S_8_ complex (Lee and Tabita, 1990), while *k*_cat_^c^ was reduced to 0.6–1% of wild-type values (Andrews, 1988; Gutteridge, 1991). More recent studies can be divided into those that have examined the impact of native RbcS mutations on Rubisco performance, and those that have attempted to assemble heterologous RbcS with native RbcL to produce a hybrid Rubisco complex. Several of these modifications have resulted in significant changes to the catalytic parameters and/or content of Rubisco (Table 2).

Chlamydomonas has been used extensively to examine the importance of different residues and features of the RbcS, in particular the variable βA–βB loop region. Spreitzer *et al.* (2001) observed that single residue substitutions in the βA–βB loop resulted in significant changes in the catalytic efficiency and specificity of Rubisco (Khrebtukova and Spreitzer, 1996; Spreitzer *et al.*, 2001). Furthermore, replacing the native βA–βB loop with a shorter variant from spinach or the model cyanobacterium *Synechococcus elongatus* PCC 7942 showed that the length of the βA–βB loop is not critical for Form IB Rubisco assembly (Karkehabadi *et al.*, 2005). Both heterologous βA–βB loops caused similar reductions in the carboxylation rate of Rubisco, while the cyanobacterial sequence resulted in a reduction in *S*_c/o_ values. These and further studies have demonstrated that changes to the βA–βB loop can impact the performance of the active sites of Rubisco even though the loop is structurally remote (i.e. >16 Å in distance), possibly through distant interactions with RbcL in the solvent pore (Spreitzer *et al.*, 2005; Esquivel *et al.*, 2013). Apart from the βA–βB loop, several highly conserved residues on the RbcS that make contact with RbcL near the β/α-barrel through van der Waals or salt bridge interactions have been shown to be important (Genkov and Spreitzer, 2009; Meyer *et al.*, 2012). For example, the substitution L18A in the RbcS N-terminal region can influence Rubisco stability, while Y32A in αA and E43A in βA impact catalytic performance.

More recent vascular plant-based studies have focused on engineering entire heterologous small subunits into host organisms to generate hybrid Rubisco complexes (Table 2). Introduction of an RbcS from C_4_*Sorghum bicolor* into rice (*Oryza sativa*) resulted in a hybrid Rubisco with more C_4_-like characteristics, including an increased *k*_cat_^c^ and decreased *S*_c/o_ (Ishikawa *et al.*, 2011; Matsumura *et al.*, 2020). Furthermore, transformation of three potato (*Solanum tuberosum*) RbcS isoforms into the chloroplast genome in tobacco (*Nicotiana tabacum*) produced hybrid Rubiscos with the native NtRbcL that had significant differences in catalysis, including a 13% and 8% increase in *k*_cat_^c^ and *S*_c/o_, respectively, for StRbcS3 (Martin-Avila *et al.*, 2020). Notably, StRbcS3 differs from two other StRbcS isoforms tested by two amino acid resides at the apex of the βA–βB loop. These results highlight the potential of modified or heterologous RbcS isoforms to enhance Rubisco performance in different crop species.

Introducing different plant RbcS variants into Chlamydomonas not only resulted in increased *S*_c/o_ values, more similar to that of higher plants, but also revealed the critical role of the algal RbcS α-helices in pyrenoid assembly (Genkov *et al.*, 2010; Meyer *et al.*, 2012). In comparison, expression of Chlamydomonas CrRbcS2 in the Arabidopsis RbcS-deficient double mutant *1a3b* (i.e. lacking expression of *AtRbcS1A* and *AtRbcS3B*) produced a hybrid Rubisco pool with reduced *S*_c/o_ values (Atkinson *et al.*, 2017). Given the evolutionary distance between plants and algae, this raises interesting questions of how broadly compatible different RbcS isoforms are for Rubisco assembly and stability in non-native species.

## RbcS regulates the content and can influence the catalytic characteristics of Rubisco in eukaryotes

In eukaryotes that possess Form IB Rubisco, RbcS is encoded by a family of nuclear-encoded genes that varies in number between species. Some species have a small RbcS gene family, such as Chlamydomonas with two isoforms, while others have much larger gene families, such as tobacco and wheat (*Triticum aestivum*) with 13 and 25, respectively (Table 3) (Donovan *et al.*, 2020; Caruana *et al.*, 2022). As the RbcS is no longer located with the RbcL on a chloroplastic operon, further regulatory processes have evolved to coordinate the efficient production of both subunits in a 1:1 stoichiometric ratio. Several studies in algae, C_3_ plants, and C_4_ plants have shown that RbcL synthesis in the chloroplast is dependent on the presence of RbcS, and that knockdown of RbcS expression reduces the rate of RbcL synthesis (Khrebtukova and Spreitzer, 1996; Rodermel *et al.*, 1996; Wostrikoff and Stern, 2007; Wostrikoff *et al.*, 2012; Khumsupan *et al.*, 2020). RbcL synthesis appears to be regulated by a process known as control by epistasis of synthesis, where in the absence of RbcS, the L_8_–RAF1 assembly intermediate (or possibly L_8_–BSD2 in species with BSD2) acts as a suppressor of RbcL translation (Wietrzynski *et al.*, 2021). Thus, RbcS expression plays a key role in regulating the size of the Rubisco pool.

Recent work has shown that increasing native RbcS expression levels can lead to improvements in growth and yield. In rice, overexpression of OsRbcS2 resulted in a 23% and 28% increase in dry weight and rice yield, respectively, compared with wild-type plants (Table 2) (Yoon *et al.*, 2020). In maize, overexpression of ZmRbcS alone had no impact on Rubisco levels (Salesse-Smith *et al.*, 2018). However, co-expression of ZmRbcS with RAF1 resulted in a 30% increase in both Rubisco content and dry weight, which suggests that RAF1 expression may co-regulate Rubisco levels and activity, at least in C_4_ plants.

### The role(s) of RbcS gene families

The expansion of gene families is driven by various gene duplication processes, at both the single-gene and whole-genome level. For example, gene duplication can occur through tandem duplications, which results in gene copies being close together on the same chromosome in a tandem array (Freeling, 2009). Tandem arrays of RbcS genes are present in many organisms, including *CrRbcS1* and *CrRbcS2* in Chlamydomonas (Goldschmidt-Clermont and Rahire, 1986)*, AtRbcS1B–AtRbcS 3B* in Arabidopsis (Krebbers *et al.*, 1988), *OsRbcS2–OsRbcS5* in rice (Morita *et al.*, 2014), five of the six RbcS genes in the facultative Crassulacean acid metabolism (CAM) plant *Mesembryanthemum crystallinum* (Derocher *et al.*, 1993), six of the eight RbcS genes in petunia (*Petunia* sp. hybrid Mitchell strain) (Dean *et al.*, 1985), and the entire RbcS gene family in pea (*Pisum sativum*) (Polans *et al.*, 1985). A further mechanism for gene duplication is transposon activity, which can transfer gene copies between different chromosomes. Transposon activity may account for the chromosomally isolated RbcS isoforms in Arabidopsis (i.e *AtRbcS1A*) and petunia. At the whole-genome level, duplication can occur through polyploidization (Van De Peer *et al.*, 2017). For example, in wheat, two allopolyploidy events have resulted in the presence of three distinct diploid subgenomes named A, B, and D, which each contributed an RbcS gene family of nine, eight, and eight genes, respectively (Table 3) (Caruana *et al.*, 2022).

Despite these duplication events, RbcS gene families generally show little sequence divergence. For example, four of the five rice RbcS genes, *OsRbcS2–OsRbc5*, encode the same mature RbcS peptide (Suzuki *et al.*, 2007). Similarly, tomato (*Solanum lycopersicum*) and Arabidopsis each encode three RbcS genes located in a tandem array that are nearly identical (Wanner and Gruissem, 1991). Given the apparent lack of sequence divergence, the evolutionary benefit of retaining individual members within RbcS gene families is somewhat unclear, and still an active area of ongoing research. One potential explanation is that multiple RbcS gene copies ensure a sufficient supply of RbcS peptides to meet the demands of Rubisco production (Khrebtukova and Spreitzer, 1996). Retaining an RbcS gene family could also help plants to respond more dynamically to a wider range of environmental and developmental stimuli. In line with the latter argument, the promoter elements regulating the expression of different RbcS genes within a family appear diverse and indicate a high degree of subfunctionalization of expression (Sugita *et al.*, 1987; Dedonder *et al.*, 1993; Cheng *et al.*, 1998; Yoon *et al.*, 2001; Sawchuk *et al.*, 2008). Furthermore, evidence for differential expression of RbcS genes in different organs and under different environmental conditions is available in several plant species including Arabidopsis, tomato, tobacco, rice, and maize (Ewing *et al.*, 1998; Narsai *et al.*, 2009; Zhang *et al.*, 2012; Laterre *et al.*, 2017; Buti *et al.*, 2018).

An alternative hypothesis is that RbcS isoforms have evolved for specific functions that impact on the catalytic properties of Rubisco. The apparent lack of sequence diversity within RbcS families argues against this hypothesis, as it is unlikely that the small differences in peptide sequences observed from current data would lead to a significant functional impact on the catalytic performance of Rubisco in photosynthetic tissues (Yamada *et al.*, 2019). Nevertheless, a broader analysis of RbcS families may reveal further complexity. For example, Sharwood *et al.* (2016) noted that the C_4_ Paniceae species *Megathyrsus maximus* and *Panicum monticola* share identical RbcL peptide sequences but showed significant differences in several catalytic parameters, suggesting that RbcS may influence Rubisco catalysis. Furthermore, *E. coli*-based characterizations of predicted ancestral Rubiscos within the Solanaceae family (i.e. tobacco and potato) have also shown that relatively small differences in the RbcS sequence can modify catalysis (Lin *et al.*, 2022). Notably, the increased abundance of mutations found in ancestral RbcS sequences indicated that the recent evolution of C_3_ Rubiscos may have been driven more by changes in the RbcS than in the RbcL.

### RbcS in the T-type cluster produce Rubisco with different catalytic properties

A new cluster of ‘T-type’ RbcS isoforms has recently been discovered that are phylogenetically distinct from RbcS isoforms expressed in leaves (i.e. M-type RbcS) (Laterre *et al.*, 2017). Initially identified in tobacco trichomes (hence ‘T’), T-type RbcS appear to have a similar tertiary structure to M-type RbcS (Fig. 2B), but differ significantly in terms of sequence homology and expression (Table 3). T-type RbcS are present in many different plant species, including pteridophytes and bryophytes, but are absent in several dicots and monocots, indicating that independent losses of T-type RbcS genes have occurred during evolution (Pottier *et al.*, 2018). Current evidence suggests that T-type RbcS facilitates Rubisco expression for non-photosynthetic processes, such as CO_2_ recycling during secondary metabolic processes (e.g. oil or fatty acid biosynthesis). Notably, Rubisco complexes assembled with T-type RbcS can have significantly different catalytic properties compared with those with M-type RbcS (Table 2). For example, Chlamydomonas Rubisco had increased *V*_c_ and *K*_c_ values when assembled with the tobacco T-type RbcS compared with an M-type NtRbcS (Laterre *et al.*, 2017). Furthermore, Rubisco carrying the T-type RbcS showed higher carboxylation rates at pH values below 8. Laterre *et al.* (2017) hypothesized that these changes may be a functional adaptation to allow Rubisco to operate in a more acidic, CO_2_-rich environment, which might be the case in specialized secretory cells compared with the more alkaline environment of the chloroplast stroma in mesophyll cells. More recently, assembly of the tobacco RbcL with different members of the RbcS family in *E. coli* has confirmed that the T-type RbcS increases the *k*_cat_^c^ and *K*_c_ values for tobacco Rubisco (Lin *et al.*, 2020). Similar changes in Rubisco catalytic performance were observed in transgenic rice plants overexpressing the rice T-type RbcS, and in transgenic tobacco plants expressing the potato RbcL and potato T-type RbcS (Morita *et al.*, 2014; Martin-Avila *et al.*, 2020).

In flowering plants, expression of T-type RbcS is limited to non-photosynthetic tissues (Voo *et al.*, 2012; Laterre *et al.*, 2017). However, in pteridophytes and bryophytes, T-type RbcS expression appears less restricted, and T-type isoforms are generally more abundant than M-type RbcS. Pottier *et al.* (2018) suggested this may be due to species in early land plant lineages having emerged before the increase of atmospheric O_2_ to modern levels (Lenton *et al.*, 2016), when Rubisco would have been adapted to a higher CO_2_ to O_2_ ratio (i.e. a higher *k*_cat_^c^ and lower *S*_c/o_). Thus, M-type RbcS could have evolved to improve the specificity of Rubisco as atmospheric O_2_ levels increased. Further measurements of the catalytic characteristics of Rubiscos with different T-type RbcS may help to uncover the past and ongoing ecological roles of the T-type cluster, and if T-type RbcS isoforms could be used to inform strategies to improve the catalytic properties of Rubiscos in crop plants.

## RbcS as an engineering target for enhancing plant photosynthesis

Despite the relatively small number of studies to date focused on overexpressing native RbcS or engineering modified or heterologous RbcS isoforms in plants, the observed wide-ranging impacts on the catalytic performance of Rubisco are encouraging (Table 2). In support of the relative ease of nuclear transformation of RbcS, recent advances in tissue culture and protoplast regeneration highlight the growing feasibility and generally robust efficiencies of nuclear genome engineering in a wide selection of crop and non-crop species (Woo *et al.*, 2015; Lin *et al.*, 2018). Research going forward should continue to focus on establishing the extent of non-native RbcS compatibility in terms of sequence and structure for expression and assembly in plants (and algae of biotechnological interest), and the range of catalytic changes achievable by the RbcS alone.

RbcS-deficient mutants of model plant species, such as tobacco or Arabidopsis (Khumsupan *et al.*, 2020; Martin-Avila *et al.*, 2020), are useful proxies to investigate the capacity of heterologous RbcS to assemble with plant RbcL, affect the catalysis of Rubisco, and impact photosynthesis and growth. *In planta* screens could also be important to confirm that an appropriate transit peptide is being used for efficient chloroplast targeting of heterologous RbcS and that appropriate promoter and terminator combinations are being employed to drive sufficient expression. This could be achieved relatively quickly through transient expression in *Nicotiana benthamiana* or by protoplast transformation of the target plant (Xu *et al.*, 2022), or an amenable close relative. More high-throughput screening approaches will probably rely on *E. coli*, which is rapidly developing as a powerful platform for expressing all Form I Rubiscos, screening RbcS and RbcL from different species, and directed evolution (Duraõ *et al.*, 2015; Aigner *et al.*, 2017; Wilson and Whitney, 2017; Lin *et al.*, 2020, 2022). Nevertheless, producing Rubiscos from plants in *E. coli* still remains challenging due to inefficient RbcL processing and the accumulation of assembly intermediates (Ng *et al.*, 2020), low functional Rubisco yields, and limited current knowledge of the chaperones required for Rubiscos from different plant species. Rubisco biogenesis in *E. coli* is also currently not a reliable predictor for assembly in chloroplasts (Wilson *et al.*, 2018), while the catalytic parameters of plant Rubiscos assembled in *E. coli* are similar but not identical to those observed *in planta* (Lin *et al.*, 2020, 2022). However, significant improvements have already been made (Zhou and Whitney, 2019), and the increasing availability of genomic data could assist with identifying homologues or additional ancillary chaperones required for different plant species (Kress *et al.*, 2022). Further progress in the state of the art of *in silico* Rubisco models should also help to complement RbcS screening approaches (Van Lun *et al.*, 2011, 2014).

A key challenge to successfully exploiting heterologous RbcS *in planta* appears to be achieving sufficiently high levels of expression in transformed plants, as production of heterologous RbcS using single expression cassettes driven by high strength promoters has not yet yielded sufficient quantities to achieve expression commensurate with native RbcS levels (Atkinson *et al.*, 2017; Matsumura *et al.*, 2020). Generating a synthetic RbcS family using a multigene expression cassette approach may help to solve this issue (Marillonnet and Grützner, 2020). Furthermore, removal or suppression of the native RbcS gene family may be required. Both CRISPR/Cas and RNAi strategies have been successfully used to remove or significantly suppress native RbcS expression levels in Arabidopsis, rice, and tobacco (Donovan *et al.*, 2020; Khumsupan *et al.*, 2020; Martin-Avila *et al.*, 2020; Matsumura *et al.*, 2020).

If improvements in Rubisco performance are achieved, other factors could become limiting to photosynthetic efficiency. For example, leaf CO_2_ diffusion is enhanced in *Limonium* species with faster Rubiscos (i.e. a higher *k*_cat_^c^) (Galmés *et al.*, 2017). Increases in CO_2_ diffusion could potentially be accomplished through the overexpression of CO_2_-permeable membrane channels, such as specific aquaporins (Kaldenhoff, 2012; Kromdijk *et al.*, 2020; Clarke *et al.*, 2022), or CO_2_/HCO_3_^−^ transporters found in algal and cyanobacterial CCMs (Rae *et al.*, 2017; Rottet *et al.*, 2021). Overexpression of chaperones could also be required, for example RAF1 or Rubisco activase in maize and rice, respectively (Salesse-Smith *et al.*, 2018; Suganami *et al.*, 2021). Furthermore, any increases in Rubisco CO_2_ assimilation may benefit from additional modifications in downstream fluxes, for example the transport of carbohydrates to sink tissues (Wang *et al.*, 2020). Modified canopy-scale models that account for Rubisco parameters may help further to predict the physiological consequences of replacing native Rubiscos with heterologous enzymes in plants (Iqbal *et al.*, 2021).

Despite these potential challenges, it is feasible that modifications to the RbcS will yield the first generation of crop plants with improved Rubiscos. RbcS changes may ultimately require concurrent modifications in the RbcL to maximize catalytic improvements or compatibility between the subunits, and this will probably become achievable as chloroplast engineering technologies improve (Yu *et al.*, 2020). Nevertheless, it is fascinating that over the past two decades the RbcS has ascended from relative obscurity to becoming a potential game changer to enhance the photosynthetic performance of plants.
